# Risk Factors for Postoperative Acute Kidney Injury in Patients Undergoing Redo Cardiac Surgery Using Cardiopulmonary Bypass

**DOI:** 10.3390/jcdd9080244

**Published:** 2022-07-29

**Authors:** Can Zhao, Yuntao Li, Guangyu Pan, Jianping Xu, Shen Liu, Yaqiong Xiao

**Affiliations:** Department of Cardiac Surgery, Peking University International Hospital, Beijing 102206, China; zhaocan2007@163.com (C.Z.); yuntaolilyt@163.com (Y.L.); guangyupanpgy@163.com (G.P.); jianpingxuxjp@163.com (J.X.)

**Keywords:** redo cardiac surgery, cardiopulmonary bypass, postoperative, acute kidney injury, risk factors

## Abstract

Objective: This paper aimed to investigate the incidence and risk factors of postoperative acute kidney injury (AKI) in adult patients undergoing redo cardiac surgery with cardiopulmonary bypass (CPB), and explore the impact of AKI on early outcomes. Methods: A total of 116 patients undergoing redo cardiac surgery with CPB between November 2017 and May 2021 were included. Patients were divided into two groups, AKI group and non-AKI group, according to the Kidney Disease Improving Global Outcomes criteria. Perioperative variables were retrospectively collected and analyzed. Risk factors for the development of AKI were investigated by univariate and multiple logistic regression models. Clinical outcomes were also compared between the groups. Results: Postoperative AKI occurred in 63 patients (54.3%), among whom renal replacement therapy was required in 12 patients (19.0%). The mechanical ventilation time (AKI: 43.00 (19.00, 72.00) hours; non-AKI: 18.00 (15.00, 20.00) hours; *p* < 0.001), ICU length of stay (AKI: 4.00 (2.00, 6.00) days; non-AKI: 3.00 (2.00, 4.00) days; *p* = 0.010), hospital length of stay since operation (AKI: 12.00 (8.00, 18.00) days; non-AKI: 9.00 (7.00, 12.50) days; *p* = 0.024), dialysis (AKI: 12.00 (19.05%); non-AKI: 0 (0%); *p* = 0.001), reintubation (AKI: 7.00 (11.11%); non-AKI: 0 (0%); *p* = 0.035), and hospital mortality (AKI: 8.00 (12.70%); non-AKI: 0 (0%); *p* = 0.020) were all higher in the AKI group than in the non-AKI group. Multivariate analysis revealed that high aspartate aminotransferase (OR, 1.028, 95% CI, 1.003 to 1.053, *p* = 0.025), coronary angiogram within 2 weeks before surgery (OR, 3.209, 95% CI, 1.307 to 7.878, *p* = 0.011) and CPB time (OR, 1.012, 95% CI, 1.005 to 1.019, *p* = 0.001) were independent risk factors for postoperative AKI. **Conclusions**: High aspartate aminotransferase, coronary angiogram within 2 weeks before surgery and CPB time seem to be associated with an increased incidence of postoperative AKI in patients with redo cardiac surgery.

## 1. Introduction

With the advances of modern medicine, the average life expectancy has been increasing at a rapid speed, leading to population aging. As a result, the demand for re-operative cardiac surgery is gradually increasing [1,2,3,4]. Despite the advanced surgical techniques and improved perioperative care, redo cardiac surgery is still marked by heightened risk and associated with major adverse events. Acute kidney injury (AKI), an independent risk factor for increased morbidity and mortality [5,6], is one of the serious complications of redo cardiac surgery [5]. Recent studies have shown that advanced age, preoperative renal dysfunction, history of diabetes mellitus, type of surgery, cardiopulmonary bypass (CPB), postoperative infection and low cardiac output are all risk factors for postoperative AKI [5,6,7]. However, the incidence and risk factors of AKI in patients undergoing redo cardiac surgery with CPB, and the influence of AKI on perioperative complications, have not yet been studied. The aim of this study was to investigate the risk factors and the impact of postoperative AKI on early outcomes in patients undergoing redo cardiac surgery.

## 2. Materials and Methods

### 2.1. Study Population

Consecutive patients receiving elective redo cardiac surgery using CPB between November 2017 and May 2021, at the Department of Cardiac Surgery of Peking University International Hospital in Beijing, China, were enrolled in the study. All the patients had previously undergone cardiac surgeries with CPB. Patients with preoperative dialysis dependence, a history of off-pump cardiac surgery, patients under the age of 18 years and patients requiring emergency surgeries were excluded from the study. A total of 146 patients were evaluated, of whom 23 had received off-pump cardiac surgeries, 5 were under the age of 18, and 2 had undergone permanent renal replacement therapy. A total of 116 patients were included in the study and were divided into two groups based on postoperative AKI.

### 2.2. Data Collection and Definitions

Medical records including demographic information, intraoperative data and postoperative data were obtained from the electronic medical record system. The fluid overload index during three days post operation was determined by the following equation [8]: (total fluid input (L)-total fluid output (L))/basic weight (kg) × 100%. The preoperative baseline serum creatinine value was defined as the most recent serum creatinine detected within 7 days before the surgery. The serum creatinine was recorded each day up to 14 days after surgery. Diagnosis and staging of postoperative AKI were based on the Kidney Disease Improving Global Outcomes (KDIGO) criteria. KDIGO 1 of AKI was diagnosed when there was an increase in serum creatinine by ≥0.3 mg/dL (≥26.5 μmol/L) within 48 h or an increase in serum creatinine to 1.5–1.9 times baseline within the previous 7 days. KDIGO 2 was diagnosed when there was an increase in serum creatinine to 2.0–2.9 times baseline. KDIGO 3 was diagnosed with 3 times baseline or ≥4.0 mg/dL (≥353.6 μmol/L) increase or initiation of renal replacement therapy. Anemia was defined as hemoglobin below 115 g/L in female patients and 130 g/L in male patients. All patients underwent continuous flow CPB. The perfusion pressure during CPB was maintained to 50–80 mmHg.

### 2.3. Statistical Analysis

Continuous variables were expressed as mean ± standard deviation and were tested using Student’s t-test if normally distributed. Non-normally distributed data were presented as medians and 25–75% interquartile ranges and were compared with the Mann–Whitney U test. Categorical variables were described as numbers and percentages and were compared with chi-square test and Bonferroni correction, continuity correction or Fisher’s exact test. The associations between potential risk factors and postoperative AKI were assessed with univariate logistic regression analysis. False-discovery rate correction was used to correct the calculated p-values. The *q*-values were calculated with Benjamini–Hochberg procedure. Significant variables with *q*-value < 0.05 were subjected to multivariable regression model. The odds ratio (OR) was calculated and presented with 95% confidence interval (CI). *p* < 0.05 was considered statistically significant for all analyses. The Kaplan–Meier survival analysis was used to explore the impact of different KDIGO stages on the 30-day postoperative mortality in the hospital. Statistical analysis was performed using SPSS software version 26.0 (IBM Corp, Armonk, NY, USA).

## 3. Results

### 3.1. Patients’ Characteristics and Postoperative AKI Incidence

One hundred and sixteen patients were included in the study, with an average age of 55.6 ± 14.6 years (20–79 years), of which 62 were female (53.45%). In their first-time cardiac surgeries, valve surgeries were performed in 96 patients, congenital cardiac surgeries in 15, cardiac tumor resections in 2, coronary artery bypass graft surgeries in 2, and surgical corrections of anomalous origin of coronary artery in 1. The average interval between the two cardiac operations was 16.1 ± 9.6 years. After redo cardiac surgery, 8 patients died in hospital, 8 suffered lung infection, 7 were reintubated, 4 received exploratory thoracotomy for bleeding, and 12 required renal replacement therapy.

Overall, 63 of the 116 patients (54.31%, 63/116) developed postoperative AKI according to the KDIGO criteria: KDIGO 1, *n* = 31 (49.21%); KDIGO 2, *n* = 16 (25.40%); KDIGO 3, *n* = 16 (25.40%). A total of 12 patients (19.0%, 12/63) required renal replacement therapy, of whom 5 (41.67%, 5/12) died in hospital. The clinical characteristics of AKI and non-AKI group are listed in Table 1. AKI occurred in 49 patients (77.78%, 49/63) on postoperative day 1, 13 (20.63%, 13/63) on postoperative day 2, and 1 (1.59%, 1/63) on postoperative day 7.

### 3.2. Comparison of Preoperative and Intraoperative Factors between AKI and Non-AKI Group

Patients with AKI had histories of pulmonary hypertension (*p* < 0.001), atrial fibrillation (*p* < 0.001) and coronary angiography within 2 weeks before surgery (*p* = 0.007) more frequently compared with those who did not have AKI. The preoperative serum creatinine (*p* = 0.018), serum uric acid (*p* = 0.018), beta-2-microglobulin (*p* < 0.001), total bilirubin (*p* = 0.008), direct bilirubin (*p* = 0.015), aspartate aminotransferase (AST) (*p* = 0.001) and B-type natriuretic peptide (*p* = 0.049) in the AKI group were all significantly higher than those without AKI, while the estimated glomerular filtration rates (eGFR) were lower than non-AKI group (*p* = 0.024). Patients in the AKI group had longer CPB duration (*p* = 0.001) and aortic cross-clamp time (*p* = 0.009), lower mean arterial pressure during CPB (*p* = 0.015), and less urine output during CPB (*p* < 0.001) and operation (*p* = 0.001) than the non-AKI group.

### 3.3. Comparison of Clinical Outcomes between AKI and Non-AKI Group

**Figure 1:**
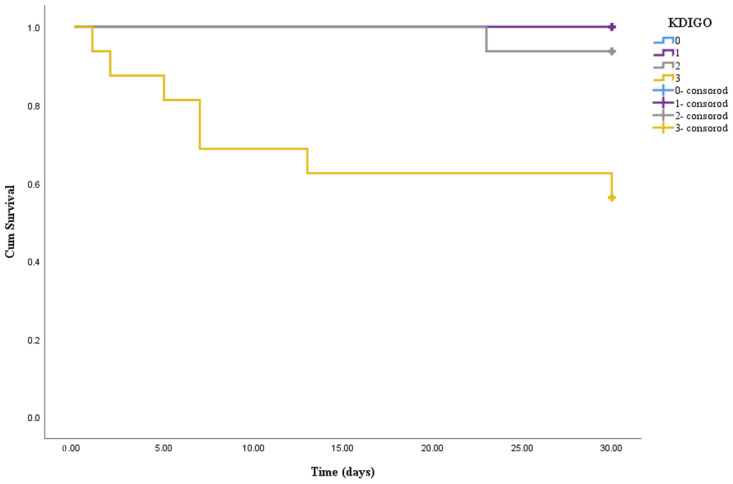
Impact of KDIGO stages on 30-day survival. Significant overall difference is observed (*p* < 0.001 by log-rank test).

Postoperative clinical data of the two groups were compared. The results showed that the mechanical ventilation time (*p* < 0.001), ICU length of stay (*p* = 0.010) and hospital length of stay since operation (*p* = 0.024) in AKI patients were longer than those without AKI; the incidence of reintubation (*p* = 0.035) and hospital mortality (*p* = 0.020) were higher than the non-AKI group. No cerebrovascular accident occurred in the two groups, and there was no significant difference in the incidence of lung infection between the two groups (*p* = 0.113). The clinical outcomes were summarized in Table 2. Kaplan–Meier survival curves (Figure 1) revealed that KDIGO stage 3 was associated with an increased 30-day mortality risk compared with stage 0–2 (chi-square: 28.58, *p* < 0.001; 16.81, *p* < 0.001; 6.16, *p* = 0.013, respectively).

### 3.4. Risk Factor Analysis of Postoperative AKI

Univariate logistic regression for postoperative AKI revealed increased age, male gender, high preoperative serum creatinine, low estimated glomerular filtration rate, elevated serum uric acid, AST, total bilirubin and direct bilirubin, preoperative pulmonary hypertension, atrial fibrillation, anemia, coronary angiogram within 2 weeks before surgery, prolonged CPB time and aortic cross clamp time, lower level of mean arterial pressure during CPB, urine output during CPB and surgery, the amount of red blood cells transfusion and platelet transfusion as risk factors (all *p* < 0.1, Table 3). In the multivariable analysis with a forward stepwise likelihood ratio method, high AST (OR, 1.028, 95% CI, 1.003 to 1.053, *p* = 0.025), coronary angiogram within 2 weeks before surgery (OR, 3.209, 95% CI, 1.307 to 7.878, *p* = 0.011) and CPB time (OR, 1.012, 95% CI, 1.005 to 1.019, *p* = 0.001) were independent risk factors for postoperative AKI (Table 4).

## 4. Discussion

The purpose of this study was to explore the incidence and risk factors of AKI defined by KDIGO standards in patients undergoing redo on-pump cardiac surgeries. Previous studies have investigated risk factors for AKI after first-time cardiac surgery with CPB, but to our knowledge, no study has explored risk factors for AKI defined by KDIGO criteria after redo cardiac surgeries [9,10,11]. In the present study, the enrolled patients all underwent redo on-pump surgery, of which valve surgery was the most performed one. We found that in addition to coronary angiogram within 2 weeks before surgery and CPB time, a high level of AST was also an independent risk factor for postoperative AKI.

The reported incidence of cardiac surgery-associated AKI varied from 1% to 48% [12,13]. This was probably due to different diagnostic criteria for AKI and different types of cardiac surgery. Recent studies have shown that the KDIGO criteria were more sensitive than the Risk, Injury, Failure, Loss, End Stage Kidney Disease (RIFLE) or Acute Kidney Injury Network (AKIN) criteria in detecting AKI [5,14,15]. In our study, the incidence of AKI diagnosed with KDIGO criteria after redo cardiac surgery was high (54.31%, 63/116). Incidence of postoperative requirement for renal replacement therapy (19.0%) in our study was also higher than the incidence reported by previous studies on first-time cardiac surgery (1.2–2.3%) [16,17]. We found that the mortality rate of patients who underwent hemodialysis was 41.7%, which was close to the incidence (40–70%) reported by Nadim et al. [15]. We also found that KDIGO stage 3 was associated with an increased 30-day mortality risk compared with stage 0–2, which was consistent with the results of previous studies that suggested increasing severity of AKI was associated with worse prognosis [17].

Both previous studies and our study have shown that older age, anemia, perioperative red blood cell transfusions, and preoperative serum creatinine level were independently associated with kidney injury [5,9,18,19]. In our study, men were more likely to develop AKI than women, which concurred with the findings of several studies [11,16,20]. It is generally acknowledged that cardiac surgery-associated AKI could be caused by insufficient fluid intake resulting in renal hypoperfusion. However, no significant difference was found in volume status within 3 days after surgery between the two groups in our study, indicating that factors other than fluid management should be considered in the prevention of AKI during the perioperative period.

Our study demonstrated that patients with preoperative pulmonary hypertension had a higher incidence rate of developing AKI. Haddad and colleagues [21] identified 105 patients with pulmonary hypertension who were hospitalized for acute right-side heart failure, finding that AKI was relatively common in patients with pulmonary hypertension, and higher central venous pressure (CVP) was associated with an increased likelihood of AKI. The mechanism behind this clinical finding might be that increased CVP leads to reduced renal perfusion pressure, impairment of renal blood flow, as well as activation of the renin–angiotensin–aldosterone and the sympathetic nervous systems, resulting in a decrease in glomerular filtration rate (GFR) [22]. Another study showed that venous congestion and decreased cardiac output in patients with pulmonary hypertension were associated with impaired kidney function [23]. A retrospective study of 1119 heart surgery patients showed that preoperative atrial fibrillation significantly increased the rate of occurrence of AKI [24]. Other studies [25,26] have come to similar conclusions, which are consistent with our results. Preoperative atrial fibrillation may lead to a low postoperative cardiac output, which further contributes to development of AKI after cardiac surgery.

Studies [27,28,29] have shown that serum uric acid is a strong predictor for AKI independent of baseline renal function. The possible mechanisms include the elevated serum uric acid leading to renal vasoconstriction, impaired autoregulation, and activation of inflammatory cascade resulting in decreased GFR. Elevated preoperative serum uric acid in cardiac surgery patients was independently associated with an increased risk for AKI after adjusting for type of surgery, preoperative serum creatinine, history of diabetes and CPB time [28]. These observations are in accordance with the results of our study. Bilirubin is the final product of heme catabolism by the liver. Excess bilirubin or hepatic dysfunction leads to hyperbilirubinemia. Our data showed that preoperative hyperbilirubinemia was a risk factor for AKI after surgery. Systemic inflammatory response, hepatic hypoperfusion and CPB-induced hemolysis during cardiac surgery may disrupt bilirubin metabolism, leading to hyperbilirubinemia. Bilirubin acts as an antioxidant at physiological concentration, but abnormally high concentrations of bilirubin can cause oxidative stress and cell apoptosis [30]. It has been suggested that hyperbilirubinemia could induce apoptosis in renal tubular epithelial cells and aggravate renal ischemia reperfusion injury [31].

Multivariate analysis revealed high AST, coronary angiogram within 2 weeks before surgery and CPB time as independent risk factors for postoperative AKI in the present study. A number of recent works [5,11,16,18,19,32,33] demonstrated that prolonged CPB time and preoperative angiography were independent risk factors for AKI. Cardiopulmonary bypass contributes to AKI because of red blood cell hemolysis and the systemic inflammatory response syndrome. A crucial point that influences these pathways is represented by the CPB time. In a recent study [19], researchers found that 91 min of CPB time was found as the threshold above which the risk for AKI started to increase significantly. One interesting finding of our study was that an increased AST was associated with an increased risk of AKI. AST is mainly found in the mitochondria of liver cells, but could also be found in the heart, skeletal muscle, lungs, brain and kidneys [34]. In extrahepatic tissues, AST is mainly released by cardiomyocytes, and myocardial injury preoperatively could lead to an increase in AST [35], which may explain the correlation between high AST and AKI, as myocardial injury is a recognized risk factor for AKI. However, alanine aminotransferase (ALT) is only present in the liver cells, which might be the reason why no correlation was found between ALT and AKI in this study. However, there are limitations of the analysis in adjusting for confounding factors. The non-linearity for the relationship between covariates and AKI should also be considered.

## 5. Limitations

This study is subject to the limitations inherent in a retrospective analysis of observational data. The validity of this study is limited because of the small samples and limited durations of clinical follow-up in a single institution, and it is insufficient to draw a definite conclusion. There might be residual bias and unconsidered confounding factors, and causality could not be determined. Secondly, only the medical records during hospitalization were collected and analyzed, and therefore, no sufficient data was available to elucidate the long-term prognosis for post-redo cardiac surgery AKI patients. Future prospective studies on a larger scale are needed to further investigate the risk factors and prognosis of postoperative AKI among redo cardiac surgery patients.

## 6. Conclusions

This study explored the preoperative and intraoperative risk factors of AKI. The incidence of postoperative AKI was high in patients with redo cardiac surgery using CPB. High AST, coronary angiogram within 2 weeks before surgery and CPB time were associated with postoperative AKI. Our findings are helpful in early identification of patients with a high risk of postoperative AKI, who may benefit from appropriate perioperative support and clinical care.

## Figures and Tables

**Table 1:** Demographics and clinical characteristics of patients undergoing redo cardiac surgery with cardiopulmonary bypass.

Characteristics	All Cases (*n* = 116)	AKI (*n* = 63)	No AKI (*n* = 53)	*p*
Age, y	57.50 (47.00, 67.80)	61.00 (55.00, 68.00)	54.00 (39.00, 67.50)	0.054
Female sex, *n* (%)	62 (53.45)	29 (46.03)	33 (62.26)	0.081
BMI, kg/m^2^	21.26 (19.20, 23.57)	21.26 (19.69, 23.80)	21.60 (18.89, 23.47)	0.549
BSA, m^2^	1.72 ± 0.16	1.72 ± 0.16	1.71 ± 0.17	0.833
Interval, y	16.07 ± 9.60	15.82 ± 8.89	16.36 ± 10.47	0.844
Diabetes (type 1 or 2), *n* (%)	13 (11.21)	6 (9.52)	7 (13.21)	0.531
Smoking, *n* (%)	8 (6.90)	4 (6.35)	4 (7.55)	1.000
Hypertension, *n* (%)	8 (6.90)	6 (9.52)	2 (3.77)	0.395
Pulmonary hypertension, *n* (%)	74 (63.79)	53 (84.13)	21 (39.62)	<0.001
Preoperative atrial fibrillation, *n* (%)	71 (61.21)	50 (79.37)	21 (39.62)	<0.001
COPD, *n* (%)	2 (1.72)	1 (1.59)	1 (1.89)	1.000
Cerebrovascular disease, *n* (%)	7 (6.03)	3 (4.76)	4 (7.55)	0.813
Peripheral vascular disease, *n* (%)	47 (40.52)	27 (42.86)	20 (37.74)	0.576
Anemia, *n* (%)	63 (54.31)	39 (61.90)	24 (45.28)	0.073
Infectious endocarditis(active), *n* (%)	7 (6.03)	5 (7.94)	2 (3.77)	0.585
Recent (<14 days) heart catheterization, *n* (%)	66 (56.90)	43 (68.25)	23 (43.40)	0.007
LVEF, %	61.52 ± 9.54	60.95 ± 9.38	62.20 ± 9.76	0.487
NYHA-3,4, *n* (%)	90 (77.59)	51 (80.95)	39 (73.58)	0.343
NYHA-2, *n* (%)	26 (22.41)	12 (19.05)	14 (26.42)	0.343
EuroSCORE II, (%)	6.68 (3.97, 11.19)	6.88 (4.11, 11.99)	5.95 (3.48, 9.75)	0.144
BNP, pg/mL	256.90 (97.50, 397.50)	305.50 (116.45, 463.85)	183.05 (78.68, 320.90)	0.049
Preoperative eGFR, mL/min/1.73 m^2^	84.25 ± 22.88	79.89 ± 20.38	89.44 ± 24.74	0.024
Baseline serum creatinine, μmol/L	75.50 (62.25, 92.00)	81.00 (66.00, 99.00)	73.00 (59.00, 82.50)	0.018
Peak serum creatinine, μmol/L	111.00 (85.25, 164.00)	150.00 (121.00, 209.00)	83.00 (67.00, 99.00)	<0.001
Preoperative urea, mmol/L	7.49 (5.98, 9.72)	7,71 (6.29, 10.75)	7.12 (5.54, 9.28)	0.159
Preoperative serum uric acid, μmol/L	423 (351.25, 561.75)	456.00 (373.00, 591.00)	387.00 (338.00, 533.00)	0.018
Preoperative beta-2-microglobulin, mg/L	2.51 (1.85, 3.59)	3.21 (2.26, 4.14)	2.13 (1.59, 2.99)	<0.001
Preoperative albumin, g/L	40.00 ± 3.66	39.97 ± 3.74	40.03 ± 3.59	0.940
Preoperative total bilirubin, μmol/L	20.90 (14.30, 35.68)	24.30 (15.10, 42.60)	17.40 (13.00, 27.40)	0.008
Preoperative direct bilirubin, μmol/L	8.95 (5.60, 14.28)	10.40 (6.10, 16.70)	7.40 (5.05, 12.10)	0.015
Preoperative alanine aminotransferase, U/L	16.00 (12.00, 23.00)	17.00 (13.00, 24.00)	15.00 (12.00, 22.50)	0.341
Preoperative aspartate aminotransferase, U/L	28.00 (22.00, 39.00)	32.00 (24.00, 54.00)	24.00 (21.00, 31.50)	0.001
CPB duration, min	185.00 (134.25, 240.25)	207.00 (148.00, 265.00)	156.00 (116.00, 208.00)	0.001
Cross-clamp time, min	123.00 (89.75, 168.25)	140.50 (98.25, 183.50)	112.50 (80.00, 152.25)	0.009
Minimum nasopharyngeal temperature, °C	31.00 (30.50, 31.68)	31.00 (30.40, 31.40)	31.00 (30.90, 32.00)	0.150
CPB-MAP, mmHg	52.02 ± 5.91	50.8 1± 5.85	53.47 ± 5.70	0.015
CPB-urine output, mL/h	84 (24, 205)	49 (18, 114)	140 (60, 268)	<0.001
Intraoperative-urine output, mL/h	121 (59, 180)	92 (46, 157)	158 (83,242)	0.001
* Fluid overload index during three days post operation, %	−4.32 (−5.79, −2.90)	−4.32 (−6.74, −2.73)	−4.31 (−5.58, −3.20)	0.969
Surgical procedure				
Valve surgery, *n* (%)	107 (92.24)	61 (96.83)	46 (86.79)	0.096
CABG, *n* (%)	3 (2.59)	0 (0)	3 (5.66)	0.185
CABG + valve surgery, *n* (%)	1 (0.86)	1 (1.59)	0 (0)	1.000
Congenital surgery, *n* (%)	4 (3.45)	1 (1.59)	3 (5.66)	0.492
Red blood cells transfusion, U	4 (2, 8)	4 (2, 10)	4 (4, 8)	0.358
Plasma transfusion, mL	600 (200, 800)	600 (200, 800)	600 (300,800)	0.888
Platelet transfusion, U	1 (0, 1)	1 (0.5, 2)	1 (0, 1)	0.055

AKI, acute kidney injury; BMI, body mass index; BSA, body surface area; COPD, chronic obstructive pulmonary disease; LVEF, left ventricular ejection fraction; NYHA, New York Heart Association (functional classification); EuroSCORE, European System for Cardiac Operative Risk Evaluation; BNP, B-type natriuretic peptide; eGFR, estimated glomerular filtration rate; CPB, cardiopulmonary bypass; MAP, mean arterial pressure; CABG, coronary artery bypass grafting. * Fluid overload index during three days post operation was determined by the following equation: (total fluid input of postoperative three days (L)-total fluid output of postoperative three days (L))/basic weight (kg) × 100%.

**Table 2:** Comparison of postoperative outcomes between patients with and without postoperative acute kidney injury.

Characteristics	AKI (*n* = 63)	No AKI (*n* = 53)	*p*
Mechanical ventilation, h	43.00 (19.00, 72.00)	18.00 (15.00, 20.00)	<0.001
ICU stay, d	4.00 (2.00, 6.00)	3.00 (2.00, 4.00)	0.010
Postoperative hospital stay, d	12.00 (8.00, 18.00)	9.00 (7.00, 12.50)	0.024
Pneumonia, *n* (%)	7.00 (11.11)	1.00 (1.89)	0.113
Re-intubation, *n* (%)	7.00 (11.11)	0 (0)	0.035
Hospital mortality, *n* (%)	8.00 (12.70)	0 (0)	0.020

ICU, intensive care unit.

**Table 3:** Univariate analysis of factors associated with postoperative acute kidney injury.

Variable	Odds Ratio	95% CI	*p*	*q*-Value *
Age	1.029	(1.002–1.056)	0.033	0.042
Female sex	0.517	(0.246–1.088)	0.082	0.082
Baseline serum creatinine	1.015	(0.998–1.031)	0.076	0.080
Preoperative eGFR	0.981	(0.964–0.998)	0.028	0.041
Preoperative uric acid	1.003	(1.000–1.005)	0.020	0.038
Preoperative total bilirubin	1.042	(1.013–1.072)	0.004	0.019
Preoperative direct bilirubin	1.085	(1.023–1.151)	0.006	0.023
Preoperative aspartate aminotransferase	1.030	(1.007–1.053)	0.011	0.026
CPB duration	1.010	(1.004–1.016)	0.001	0.019
Cross-clamp time	1.011	(1.003–1.018)	0.007	0.022
CPB-MAP	0.924	(0.865–0.986)	0.017	0.036
CPB-urine output	0.995	(0.991–0.998)	0.002	0.019
Intraoperative-urine output	0.993	(0.989–0.998)	0.002	0.013
Pulmonary hypertension	2.726	(1.127–6.592)	0.026	0.045
Preoperative atrial fibrillation	2.571	(1.061–6.234)	0.037	0.044
Anemia	1.964	(0.935–4.125)	0.075	0.084
Recent (<14 days) heart catheterization	2.804	(1.312–5.992)	0.008	0.022
Red blood cells transfusion	1.088	(1.007–1.174)	0.032	0.043
Platelet transfusion	1.790	(1.070–2.996)	0.027	0.043

CI, confidence interval; eGFR, estimated glomerular filtration rate; CPB, cardiopulmonary bypass; MAP, mean arterial pressure. * The q-values were false-discovery rate correction of *p*-values.

**Table 4:** Multivariable analysis of factors associated with postoperative acute kidney injury.

Variable	Odds Ratio	95% CI	*p*
Preoperative aspartate aminotransferase	1.028	1.003–1.053	0.025
CPB duration	1.012	1.005–1.019	0.001
Recent (<14 days) heart catheterization	3.209	1.307–7.878	0.011

CI, confidence interval; CPB, cardiopulmonary bypass.

## Data Availability

The original contributions presented in the study are included in the article, and further inquiries can be directed to the corresponding author.

## References

[1] Bianco V., Kilic A., Gleason T.G., Aranda-Michel E., Habertheuer A., Wang Y., Navid F., Kacin A., Sultan I. (2020). Reoperative Cardiac Surgery Is a Risk Factor for Long-Term Mortality. Ann. Thorac. Surg..

[2] Kilic A., Arnaoutakis G.J., Bavaria J.E., Sultan I., Desai N.D., Vallabhajosyula P., Williams M.L., Milewski R.K., Szeto W.Y. (2017). Outcomes of elective aortic hemiarch reconstruction for aneurysmal disease in the elderly. Ann. Thorac. Surg..

[3] Zakkar M., Bruno V.D., Guida G., Angelini G.D., Chivasso P., Suleiman M.S., Bryan A.J., Ascione R. (2016). Postoperative acute kidney injury defined by RIFLE criteria predicts early health outcome and long-term survival in patients undergoing redo coronary artery bypass graft surgery. J. Thorac. Cardiovasc. Surg..

[4] Kilic A., Acker M.A., Gleason T.G., Sultan I., Vemulapalli S., Thibault D., Ailawadi G., Badhwar V., Thourani V., Kilic A. (2019). Clinical outcomes of mitral valve reoperations in the United States: An analysis of the Society of Thoracic Surgeons National Database. Ann. Thorac. Surg..

[5] Wang Y., Bellomo R. (2017). Cardiac surgery-associated acute kidney injury: Risk factors, pathophysiology and treatment. Nat. Rev. Nephrol..

[6] Tseng P.-Y., Chen Y.-T., Wang C.-H., Chiu K.-M., Peng Y.-S., Hsu S.-P., Chen K.-L., Yang C.-Y., Lee O.K.-S. (2020). Prediction of the development of acute kidney injury following cardiac surgery by machine learning. Crit. Care.

[7] Yi Q., Li K., Jian Z., Xiao Y.-B., Chen L., Zhang Y., Ma R.-Y. (2016). Risk Factors for Acute Kidney Injury after Cardiovascular Surgery: Evidence from 2,157 Cases and 49,777 Controls-A Meta-Analysis. Cardiorenal Med..

[8] Seguin J., Albright B., Vertullo L., Lai P., Dancea A., Bernier P.-L., Tchervenkov C.I., Calaritis C., Drullinsky D., Gottesman R. (2014). Extent, risk factors, and outcome of fluid overload after pediatric heart surgery*. Crit. Care Med..

[9] Karkouti K., Wijeysundera D., Yau T.M., Callum J.L., Cheng D.C., Crowther M., Dupuis J.-Y., Fremes S., Kent B., Laflamme C. (2009). Acute kidney injury after cardiac surgery: Focus on modifiable risk factors. Circulation.

[10] Yamauchi T., Miyagawa S., Yoshikawa Y., Toda K., Sawa Y. (2017). Osaka cardiovascular surgery research (OSCAR) group. risk index for postoperative acute kidney injury after valvular surgery using cardiopulmonary bypass. Ann. Thorac. Surg..

[11] Fu H.-Y., Chou N.-K., Chen Y.-S., Yu H.-Y. (2021). Risk factor for acute kidney injury in patients with chronic kidney disease receiving valve surgery with cardiopulmonary bypass. Asian J. Surg..

[12] Parolari A., Pesce L., Pacini D., Mazzanti V., Salis S., Sciacovelli C., Rossi F., Alamanni F. (2012). Risk factors for perioperative acute kidney injury after adult cardiac surgery: Role of perioperative management. Ann. Thorac. Surg..

[13] Hobson C.E., Yavas S., Segal M.S., Schold J.D., Tribble C.G., Layon A.J., Bihorac A. (2009). Acute kidney injury is associated with increased long-term mortality after cardiothoracic surgery. Circulation.

[14] Khwaja A. (2012). KDIGO clinical practice guidelines for acute kidney injury. Nephron Clin. Pract..

[15] Nadim M.K., Forni L.G., Bihorac A., Hobson C., Koyner J.L., Shaw A., Arnaoutakis G.J., Ding X., Engelman D.T., Gasparovic H. (2018). Cardiac and Vascular Surgery-Associated Acute Kidney Injury: The 20th International Consensus Conference of the ADQI (Acute Disease Quality Initiative) Group. J. Am. Heart Assoc..

[16] Xie X., Wan X., Ji X., Chen X., Liu J., Chen W., Cao C. (2017). Reassessment of Acute Kidney Injury after Cardiac Surgery: A Retrospective Study. Intern. Med..

[17] Hu J., Chen R., Liu S., Yu X., Zou J., Ding X. (2016). Global Incidence and Outcomes of Adult Patients with Acute Kidney Injury After Cardiac Surgery: A Systematic Review and Meta-Analysis. J. Cardiothorac. Vasc. Anesth..

[18] Leballo G., Chakane P.M. (2020). Cardiac surgery-associated acute kidney injury: Pathophysiology and diagnostic modalities and management. Cardiovasc. J. Afr..

[19] Serraino G.F., Provenzano M., Jiritano F., Michael A., Ielapi N., Mastroroberto P., Andreucci M., Serra R. (2021). Risk factors for acute kidney injury and mortality in high risk patients undergoing cardiac surgery. PLoS ONE.

[20] Freeland K., Jahromi A.H., Duvall L.M., Mancini M.C. (2015). Postoperative blood transfusion is an independent predictor of acute kidney injury in cardiac surgery patients. J. Nephropathol..

[21] Haddad F., Fuh E., Peterson T., Skhiri M., Kudelko K.T., Perez V.D.J., Winkelmayer W.C., Doyle R.L., Chertow G.M., Zamanian R.T. (2011). Incidence, correlates, and consequences of acute kidney injury in patients with pulmonary arterial hypertension hospitalized with acute right-side heart failure. J. Card. Fail..

[22] Damman K., Voors A.A., Navis G., van Veldhuisen D.J., Hillege H.L. (2011). The cardiorenal syndrome in heart failure. Prog. Cardiovasc. Dis..

[23] Damman K., Navis G., Smilde T.D., Voors A.A., Van Der Bij W., Van Veldhuisen D.J., Hillege H.L. (2007). Decreased cardiac output, venous congestion and the association with renal impairment in patients with cardiac dysfunction. Eur. J. Heart Fail..

[24] Anghel D., Anghel R., Corciova F., Enache M., Tinica G. (2014). Preoperative arrhythmias such as atrial fibrillation: Cardiovascular surgery risk factor. Biomed. Res. Int..

[25] Kristovic D., Horvatic I., Husedzinovic I., Sutlic Z., Rudez I., Baric D., Unic D., Blazekovic R., Crnogorac M. (2015). Cardiac surgery-associated acute kidney injury: Risk factors analysis and comparison of prediction models. Interact. Cardiovasc. Thorac. Surg..

[26] Haase-Fielitz A., Haase M., Bellomo R., Calzavacca P., Spura A., Baraki H., Kutschka I., Albert C. (2017). Perioperative Hemodynamic Instability and Fluid Overload are Associated with Increasing Acute Kidney Injury Severity and Worse Outcome after Cardiac Surgery. Blood Purif..

[27] Ejaz A.A., Johnson R.J., Shimada M., Mohandas R., AlQuadan K.F., Beaver T.M., Lapsia V., Dass B. (2019). The Role of Uric Acid in Acute Kidney Injury. Nephron.

[28] Kaufeld T., Foerster K.A., Schilling T., Kielstein J.T., Shrestha M., Haller H.G., Haverich A., Schmidt B.M.W. (2018). Preoperative serum uric acid predicts incident acute kidney injury following cardiac surgery. BMC Nephrol..

[29] Ejaz A.A., Beaver T.M., Shimada M., Sood P., Lingegowda V., Schold J.D., Kim T., Johnson R.J. (2009). Uric acid: A novel risk factor for acute kidney injury in high-risk cardiac surgery patients?. Am. J. Nephrol..

[30] Asad S., Singh S., Ahmad A., Khan N.U., Hadi S. (2001). Prooxidant and antioxidant activities of bilirubin and its metabolic precursor biliverdin: A structure-activity study. Chem. Biol. Interact..

[31] Yuan L., Liao P.-P., Song H.-C., Zhou J.-H., Chu H.-C., Lyu L. (2019). Hyperbilirubinemia Induces Pro-Apoptotic Effects and Aggravates Renal Ischemia Reperfusion Injury. Nephron.

[32] Dayan V., Stanham R., Soca G., Genta F., Mariño J., Lorenzo A. (2017). Early surgery after angiography in patients scheduled for valve replacement. Asian Cardiovasc. Thorac. Ann..

[33] Garcia S., Ko B., Adabag S. (2012). Contrast-induced nephropathy and risk of acute kidney injury and mortality after cardiac operations. Ann. Thorac. Surg..

[34] Woreta T.A., Alqahtani S. (2014). Evaluation of abnormal liver tests. Med. Clin. N. Am..

[35] Ewid M., Sherif H., Allihimy A.S., Alharbi S.A., Aldrewesh D.A., Alkuraydis S.A., Abazid R. (2020). AST/ALT ratio predicts the functional severity of chronic heart failure with reduced left ventricular ejection fraction. BMC Res. Notes.

